# The safety of valproic acid treatment in children with epilepsy: a retrospective real-world research

**DOI:** 10.1186/s42494-024-00188-x

**Published:** 2024-12-06

**Authors:** Xiner Chen, Yujie Zhang, Xinan Liu, Ruoyu Tan, Dezhi Cao, Li Chen, Yan Hu, Bing Li, Tieshuan Huang, Qiang Zhou, Jialun Wen, Jianxiang Liao

**Affiliations:** 1 Department of Pediatrics, Shenzhen Children’s HospitalAffiliated to, China Medical University , Shenzhen, 518038 China; 2 Pediatric Neurology, Shenzhen Children’s Hospital, Shenzhen, 518038 China; 3 Guangdong Provincial Key Laboratory of Brain Connectome and Behavior, CAS Key Laboratory of Brain Connectome and Manipulation, Brain Cognition and Brain Disease Institute (BCBDI), Shenzhen Institute of Advanced Technology, Chinese Academy of Sciences, Shenzhen, 518055 China; 4 Shenzhen Graduate School, Peking University, University Town of Shenzhen, Nanshan District, Xili, Shenzhen, 518055 China

**Keywords:** Real-world research, Valproic acid, Adverse reaction, Epilepsy, Children

## Abstract

**Background:**

Liver damage, coagulopathy, hyperammonemia, fracture, menstrual disorder and amenorrhea are the most concerned adverse drug reactions of valproic acid (VPA). This study was aimed to retrospectively investigate the incidence of adverse drug reactions of VPA in the real world and its association with the age of patients and duration of treatment in order to obtain the safety data of VPA in children with epilepsy.

**Methods:**

A total of 1943 patients diagnosed as epilepsy by the Pediatric Neurology Department of Shenzhen Children’s Hospital between December 2013 and December 2023, were included in the study. They received VPA as an initial treatment, and had followed up examinations over a time span of at least two years focusing on the adverse drug reactions of VPA.

**Results:**

There was no significant difference in the incidence of liver damage, coagulation test abnormalities, and nasal bleeding during VPA monotherapy (30–90 days, 90–180 days, and > 2 years). Adolescent female patients (first visit age ≥ 12 years) showed no significant difference in the incidence of adverse reactions and abnormal ultrasound of the reproductive system pre- versus post-treatment at the first visit, similar for those below 12 years. However, laboratory blood tests revealed significantly age-dependent changes in certain biochemical markers. Two patients stopped VPA treatment due to thrombocytopenia and ovarian cystic mass comorbid with endometrial hyperplasia, recovering after VPA withdrawal.

**Conclusions:**

The initial monotherapy of VPA is generally safe in children with epilepsy of all age ranges. In the real world, VPA does not increase the risk of liver damage, coagulation disorder, elevated blood ammonia, fractures, or low serum sodium, but may significantly decrease the platelet count at 3, 6, 12, and 24 months of treatment. There is no evidence showing that VPA may increase the incidence of impairment of adolescent female reproductive system. Among children under 1 year old, it is recommended to monitor the levels of serum ammonia and aspartate aminotransferase carefully.

**Trial registration:**

ChiCTR2300075115.

## Background

Epilepsy is a common nervous system disease. Excessive discharges of neurons due to a variety of complex causes lead to recurrent, paroxysmal and transient dysfunction of the central nervous system. Epilepsy has a higher frequency in children than in adults [1, 2]. Valproic acid (VPA) is one of the commonly used broad-spectrum anti-seizure medications (ASMs) in clinical practice, often used as an initial treatment and monotherapy in the real world [3]. Therefore, its adverse drug reactions (ADRs) have always been a concern among medical practitioners [4]. VPA-related ADRs are reported to involve multiple aspects, such as liver injury, hyperammonemia, osteoporosis, hyponatremia, and thrombocytopenia [5–7]. In addition, studies have shown that females receiving VPA treatment have a higher incidence of polycystic ovary syndrome than those receiving other ASMs [8]. However, there is a certain gap between literature, guidelines, and clinical practice. In this study, we set out to explore the safety of VPA treatment in childhood epilepsy and examine the correlation between the ADRs of VPA and treatment population, age, and duration, in order to provide better guidance for rational drug use in clinics.

## Methods

### Patients

Children diagnosed with epilepsy in the Pediatric Neurology Department of Shenzhen Children’s Hospital from December 2013 to December 2023, were included in this study. They received VPA as an initial treatment and had follow-up examinations over a time span of at least 2 years. The inclusion criteria were: (1) a diagnosis with epilepsy; (2) receiving VPA as an initial treatment; (3) had follow-up examinations for over 24 months; (4) aged 0–18 years at the first visit; (5) having no significant missing or errors of clinical recording.

### Methods

Information of sex, age at the first visit, time of follow up, diagnostic data (including primary and other diagnoses), the time of first dose of VPA, the duration of VPA treatment, the medical record (including the record of out-patient and emergency treatment, admission and discharge, and the courses), and auxiliary examination results of all eligible patients was collected from the epilepsy clinical data platform of the Shenzhen Children’s Hospital.

### The VPA dosage and ADR evaluation

The conventional dosage of VPA was 20–30 mg/kg, divided twice daily. The trough plasma concentration of VPA was 50–100 mg/l during therapeutic drug monitoring. The causal correlation with ADRs was classified to be positive, likely, or possible [9].

### Observational targets

1. ADRs indicated from diagnostic information, including attention deficits, nosebleeds, liver damage, etc. Attention deficits were evaluated with standard procedures when there were complaints of attention or learning problems from the patient’s family [10, 11].
2. ADRs indicated from auxiliary examinations, including abnormal results of reproductive ultrasound examination, elevated liver enzymes, and elevated serum ammonia levels.

### Statistical methods

In this retrospective study, statistical analysis was conducted using the SPSS 27.0 software. Quantitative data regarding numbers of patients and/or percentages were tested by χ^2^ test or Fisher’s exact test. Qualitative data which did not follow normal distribution are presented as the median and interquartile range. There was a high correlation and independence between the test results of repeated measurements, and the difference between the groups were analyzed using linear mixed model. The difference is considered statistically significant when *P* < 0.05.

## Results

### The distribution of VPA initial treatment

Between December 2013 and December 2023, a total of 1943 children with epilepsy received initial VPA treatment in the Neurology Department of Shenzhen Children’s Hospital and had follow-up examinations in the hospital over a span of at least 24 months (including inpatient and outpatient visits). Their age at first visit was 0–216 months (mean ± standard deviation: 70.20 ± 50.49, median 63), and 65% were males. Of these patients, 1705 received VPA initial treatment for over 30 days (with a retention rate of 87.75%), 1648 received VPA initial treatment for over 90 days (with a retention rate of 84.82%), 1596 patients received VPA initial treatment for over 180 days (with a retention rate of 82.14%), and 740 patients (487 males and 253 females) received VPA long-term monotherapy (over two years). The age at first visit of  the patients in this study was mainly at 3 years old and above (n = 492, 66%).

**Fig. 1:**
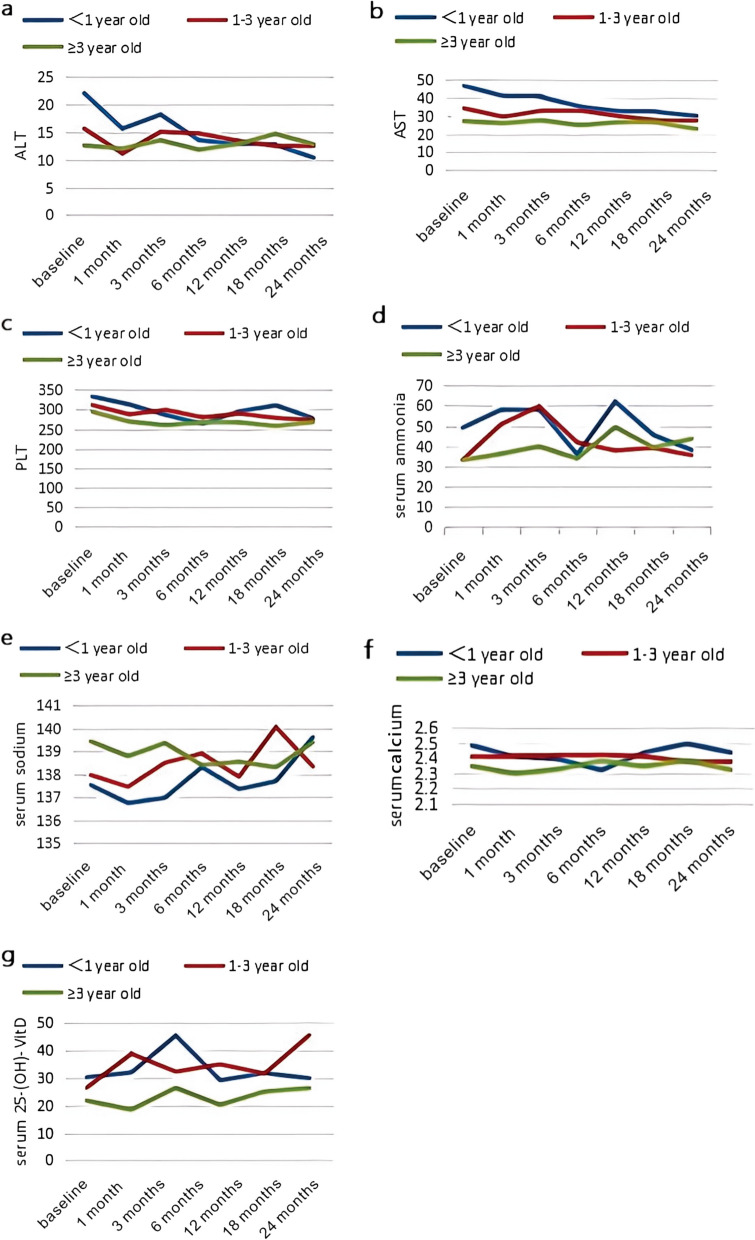
The abnormality rate of laboratory indexes in different age groups after long term VPA; VPA: Valproic acid. **a-g** different test trends. **a** the trend of variation of ALT levels in different age groups. **b** the AST levels decreased as age increased. *P* < 0.001. **c** the trend of variation of PLT counts in different age groups. **d** the serum ammonia levels decreased as age increased. *P* = 0.015. **e** the trend of variation of serum sodium levels in different age groups. **f** the levels of serum calcium decreased as age increased. *P* = 0.015. **g** the trend of variation of 25-(OH)-VitD levels in different age group

In detail, 57 patients received VPA treatment for 30–90 days and had follow-up examinations for more than two years, including 6, 11, 18 and 22 patients with age at first visit of 0–11 months, 12–35 months, 36–83 months, and over 84 months; 52 patients received VPA treatment for 90–180 days and had follow-up examinations for more than two years, including 13, 5, 18 and 16 patients with age at first visit of 0–11 months, 12–35 months, 36–83 months, and over 84 months; and 740 patients received two years of VPA monotherapy, including 97, 150, 227 and 265 patients with age at first visit of 0–11 months, 12–35 months, 36–83 months, and over 84 months.( Fig. 1)

### Effects of short-term VPA initial treatment on liver function and blood coagulation

The results of liver function and blood coagulation function in different groups are shown in Tables 1 and 2. Fisher’s exact test showed that there were no significant differences in the positive rates of liver damage, blood coagulation abnormalities, diagnosed liver function damage and diagnosed nasal bleeding among the three groups. A 1-year-old boy with West syndrome had a minimum platelet (PLT) count of 28*10^9/L after VPA treatment for 3 months, but no bleeding was reported. The maximum daily dose of VPA was 360 mg daily. The thrombocytopenia was attributed to the VPA, as the PLT count returned back to normal (205 * 10 ^ 9 / L) nine days after stopping VPA treatment. PLT antibody was negative.

**Table 1:** The positive rate of liver damage and blood coagulation in short term VPA initial treatment group and long term (over 2 years) VPA monotherapy group (%)

Adverse drug reactions	Short term treatment		Long term monotherapy	*P*-value
	30–90 days	90–180 days		
Liver damage (tests)	5 (26.32)	4 (22.22)	39 (14.44)	0.224
Abnormal blood coagulation function	1 (4.00)	0 (0.00)	0 (0.00)	0.166
Diagnosed liver damage	0 (0.00)	1 (1.92)	4 (0.54)	0.293
Diagnosed nasal bleeding	1 (1.75)	1 (1.92)	14 (1.89)	0.218

*VPA* Valproic acid

**Table 2:** ADRs of long-term VPA monotherapy in children with epilepsy (*n* = 740)

ADRs	Patients with positive ADRs (*n*)	Before treatment (%)	After treatment (%)
Attention defect	33	18 (2.43)	15 (2.03)
Nasal bleeding	16	2 (0.27)	14 (1.89)
Fracture	15	4 (0.54)	11 (1.48)
Enuresis	9	3 (0.41)	6 (0.81)
Hyperammonemia	7	1 (0.14)	6 (0.81)
Liver damage	4	0 (0.00)	4 (0.54)
Abnormal uterine bleeding	3	1 (0.40)	2 (0.80)
Amenorrhea	1	0 (0.00)	1 (0.40)
Menstrual disturbance	1	1 (0.40)	0 (0.00)

ADRs of abnormal uterine bleeding, amenorrhea and menstrual disturbance’s incidence in 253 female patients

*n* number, *ADRs* adverse drug reactions, *VPA* valproic acid

### The ADRs of VPA initial treatment on adolescent female reproductive system

Forty adolescent female patients (aged ≥ 12 at first visit) receiving VPA initial treatment underwent ultrasound examination of the reproductive system. Among these patients, five patients developed ovarian cystic mass: one of them had menstrual disorder before the VPA treatment and was excluded from ADR patients; one patient had comorbid endometrial hyperplasia, who recovered after VPA discontinuation. The incidence of menstrual disorder or dysmenorrhea and ovarian cystic mass did not statistically differ before versus after the VPA treatment (*P* > 0.05, Table 3).

**Table 3:** Reproductive system abnormalities in adolescent female epilepsy patients before and after VPA initial treatment (*n* = 40)

Symptom/examination	VPA initial treatment		*P* value
	Before	After	
Menstrual disorder or dysmenorrhea	1 (5.0)	5 (20.0)	0.205
Ovarian cystic mass	2 (9.5)	4 (15.4)	0.678

*VPA* valproic acid

### ADRs during long-term (over two years) VPA monotherapy

#### ADRs in clinical diagnostic data

The details of ADRs collected from the diagnostic data are shown in Table 2. The incidence of attention defects after long-term VPA treatment was lower than that before the treatment, while nasal bleeding, fractures, hyperammonemia and liver damage all had higher incidence than before the treatment. After the VPA treatment, two patients had fractures, two patients had abnormal uterine bleeding (females, aged < 12 at first visit, 0.9% (2/234)), and one patient had amenorrhea (female, aged ≥ 12 at first visit, 5.3% (1/19)); there was no statistical difference in the incidence of reproductive system abnormalities between the first-visit age ≥ 12 group and the first-visit age < 12 group (*P* = 0.210).

#### ADRs incidence from laboratory data

Laboratory test data on alanine aminotransferase (ALT), spartate aminotransferase (AST), serum ammonia, serum sodium, serum calcium, 25-(OH)-VitD, and PLT were collected from 263, 270, 162, 176, 187, 49, and 315 patients, respectively. Comparisons of the results before versus after the treatment are shown in Table 4. Comparisons of the results among different groups of first-visit age are shown in Table 5. Only 25-(OH)-VitD showed significant abnormality after the treatment compared to baseline (*P* < 0.05). The AST abnormality rate in the < 1 year group was significantly higher than those in other age groups (*P* < 0.017), while no statistical difference was found between the 1–3 years group and the ≥ 3 years group (*P* > 0.017). The abnormality rate of serum ammonia was significantly higher in the < 1 year group than in the ≥ 3 years group (*P* < 0.017). The abnormality rate of serum calcium was significantly higher in the ≥ 3 years group than the 1–3 years group, while no statistically significant difference was found in the first-visit age < 1 year group compared to all other groups (*P* > 0.017).

**Table 4:** Laboratory data before and after long-term VPA monotherapy treatment (%)

Laboratory items	Baseline (%)	During treatment (%)	*P*-value
ALT > 40 IU/L	3 (2.2)	4 (1.9)	1.000
AST > 40 IU/L	14 (10.3)	27 (12.9)	0.471
Serum ammonia > 72 μmol/L	5 (5.2)	10 (11.9)	0.105
Serum sodium < 135 mmol/L	3 (3.0)	6 (6.3)	0.324
Serum calcium < 2.2 mmol/L	8 (7.2)	12 (11.7)	0.265
25-(OH)-VitD < 30 ng/ml	21 (87.5)	15 (55.6)	0.012*
PLT < 150 × 10^9^/L	4 (2.5)	11 (4.4)	0.305

*VPA* Valproic acid, *ALT* Alanine aminotransferase, *AST* Aspartate aminotransferase, *25-(OH)-VitD* 25 hydroxy-vitamin D, *PLT* Platelet

^*^(*P* < 0.05)

**Table 5:** Laboratory data in different first-visit age groups after over two years of VPA monotherapy treatment (%)

Laboratory items	Age at first visit			*P-*value
	< 1 year	1–3 years	≥ 3 years	
ALT > 40 IU/L	0 (0.0)	0 (0.0)	4 (3.3)	0.485
AST > 40 IU/L	15 (34.9)^a、b^	4 (8.9)^a^	8 (6.6)^b^	< 0.001
Serum ammonia > 72 μmol/L	6 (30.0)^c^	2 (9.1)	2 (4.8)^c^	0.015
Serum sodium < 135 mmol/L	3 (13.0)	1 (4.2)	2 (4.0)	0.357
Serum calcium < 2.2 mmol/L	1(4.3)	0 (0.0)^d^	11 (20.0)^d^	0.015
25-(OH)-VitD < 30 ng/ml	3 (37.5)	3 (42.9)	9 (75.0)	0.227
PLT < 150 × 10^9^/L	1 (2.0)	2 (3.4)	6 (4.2)	0.900

① The three age groups were compared in pairs and was considered to have statistical significance when *P* < 0.017.② *Pa* = 0.003, *Pb* < 0.001, *Pc* = 0.011, *Pd* = 0.014, statistical significance was found

*VPA* Valproic acid, *ALT* Alanine aminotransferase, *AST* Aspartate aminotransferase, *25-(OH)-VitD* 25 hydroxy-vitamin D, *PLT* Platelet

### Variation of the laboratory test results among different time points of VPA treatment and different ages at first visit

For patients receiving over two years of VPA monotherapy, laboratory test results at baseline (before treatment), 1 month, 3 months, 6 months, 12 months, 18 months, and 24 months of treatment were compared. These patients were divided into < 1 year, 1–3 years, and ≥ 3 years groups according to the age at first visit. There was no statistically significant change in the levels of ALT, AST, serum ammonia, serum sodium, serum calcium and 25-(OH)-VitD at various time points compared to the baseline (*P* > 0.05). After 3 months, 6 months, 12 months and 24 months of long-term VPA monotherapy treatment, the PLT counts were significantly decreased compared to their baseline values (*P* < 0.05). Nonetheless, the PLT counts were still within the normal range, and none of the patients had a PLT count < 100 × 10^9^/L. After the long-term VPA monotherapy, the serum calcium level in the ≥ 3 years group was significantly lower than that in the < 1 year group (*P* < 0.05), while there was no significant difference in serum ammonia, serum sodium and 25-(OH)-VitD between different age groups (*P* > 0.05).

## Discussion

Epilepsy is a chronic brain disease caused by multiple etiologies. Currently, drug therapies remain the main option for its treatment. In this real-world study, we retrospectively reviewed data on ADRs associated with VPA treatment in children with epilepsy. Results showed that the initial VPA monotherapy is generally safe for children with epilepsy of all age groups. VPA did not increase the risk of liver damage, coagulation disorder, elevated blood ammonia, fractures, or low serum sodium. Nor did it increased the incidence of adverse effects in the reproductive system of adolescent females, providing a basis for the safety of clinical application of VPA.

With regard to liver damage, this study showed that neither short-term nor long-term VPA monotherapy had any significant impact. Haznedar et al. [12] did not observe any evidence of hepatotoxicity in epilepsy patients receiving therapeutic dosages of VPA. In our study, the rate of AST abnormality after VPA treatment was significantly higher in patients with an age < 1 year at first visit than the other age groups, but there was no significant difference between patients with an age of 1–3 years and those with an age ≥ 3 years at first visit. According to literature, while children of all ages are at risk of liver damage, infants and young children appear to be at a higher risk and may even result in death [13, 14]. Therefore, the margin of safety for young children to receive VPA treatment may be wider in the real world.

With regard to blood coagulation, we found that at various time points (3–24 months) of long-term VPA monotherapy, the PLT counts significantly decreased but remained within the normal range. In addition, there was no significant difference in the incidence of nasal bleeding between the short-term and the long-term VPA monotherapy groups. VPA-induced thrombocytopenia may occur several months after treatment, yet it appears to have no clinical manifestations [15]. There have also been reports of severe cerebral hemorrhage and diffuse pulmonary hemorrhage caused by VPA [16]. Therefore, further research is required to determine whether patients receiving VPA treatment should discontinue VPA before surgery.

With regard to ammonia and electrolytes, we found that the long-term VPA monotherapy did not significantly increase the incidence of hyperammonemia, but the probability of hyperammonemia occurring in children with first-visit age under 1 year was significantly higher than that in children with first-visit age ≥ 3 years. According to previous reports [17, 18], young age is one of the risk factors for hyperammonemia, and over 50% of hyperammonemia cases caused by VPA have no clinical symptoms. The possibility of global developmental delay caused by chronic hyperammonemia in children should not be ignored. Therefore, it is necessary to reinforce the monitoring of blood ammonia, especially during infancy. Confirming accuracy of laboratory error of assay was the first step when the test results was abnormal. In addition, this study found that the long-term VPA monotherapy had no significant effect on blood sodium, confirming that hyponatremia is a rare ADR induced by VPA [19].

With regard to bone metabolism, 14 patients in this study had fractures after long-term VPA monotherapy, with a lower positive rate (1.8%) than that of primary and middle school students in the city (2.4%) [20]. In addition, after the long-term VPA monotherapy, there was no significant decrease in 25-(OH)-VitD and blood calcium levels compared to that before the treatment. As multiple studies have shown [21–23] that long-term VPA monotherapy can lead to osteoporosis, the negative effects of VPA on bones can be reduced by Vitamin D supplementation. In our real-world cohort, over 100 patients had followed expert recommendations to take supplemental Vitamin D and calcium.

In addition, this study found that the ADRs on the reproductive system of adolescent females induced by the initial VPA treatment were mainly manifested as menstrual disorders and ultrasound suggested ovarian cystic masses, yet the overall incidence did not increase significantly. A study found that females have an increased incidence of menstrual disorders and polycystic ovary syndrome after VPA treatment, which is related with the hyperandrogenism caused by the metabolism of VPA [24]. As the number of patients in this study was small and the sex hormones were not synchronous monitored, further studied are needed to determine the impact of VPA on the reproductive system of adolescent females. The package insert of VPA particularly states the side effects of attention deficits that might occur mainly in the population of pediatric users. However, in this study, the incidence rate of attention deficits was not increased in patients with long-term VPA monotherapy.

Our study had limitations. First, this was a real-world retrospective study, and information was not complete. In addition, requirement of time span of visits at least two years long led to exclusion of other patients with VPA monotherapy, thus there might be a selection bias. The advantage of this study was that it was conducted in a large-scale, comprehensive pediatric epilepsy center, it included all patients’ data in line with the inclusion criteria, that was based on the medical record and to large extent reflect the overall situation, and therefore has a unique scientific value.

VPA is a traditional anti-seizure medication with global application for many years, but there remains concerns on its safety. This study used ten years’ real-world data from a pediatric comprehensive epilepsy center, and was based on the information system of hospital medical records. Our study reported the safety of VPA as an initial monotherapy in children of different ages, suggesting that VPA could still be used as a common basic drug. Future prospective studies are needed to strengthen the monitoring of endocrine functions, in order to better understand the safety of VPA.

## Conclusions

The short-term and long-term VPA monotherapy is generally safe for children with epilepsy of all ages. In the real world, treatment with conventional dosage of VPA does not increase the risk of liver damage, coagulation disorders, elevated blood ammonia, decreased serum sodium, fractures, and ADRs on the reproductive system of adolescent females. Nonetheless, the infants may have a higher incidence of abnormal serum ammonia and AST, and the PLT count decreased significantly at 3, 6, 12, and 24 months in this study. Despite the fact that it is not related to the administration of VPA, it is recommended to maintain regular monitoring in order to make personalized adjustments to medication.

## Data Availability

The datasets of the current study are available from the corresponding author on reasonable request.

## Abbreviations

ADRs: Adverse drug reactions
ALT: Alanine aminotransferase
ASMs: Anti-seizure medications
AST: Aspartate aminotransferase
25-(OH)-VitD: 25 Hydroxy-vitamin D
PLT: Platelet
VPA: Valproic acid
